# The Hardest Mode to Die

**Published:** 1876-11

**Authors:** 


### THE HARDEST MODE TO DIE.

To be shot dead is one of the easiest modes of terminating life; yet, rapid as it is, the body has leisure to feel and reflect. On the first attempt by one of the frantic adherents of Spain, to assassinate William, Prince of Orange, who took the lead in the revolt of the Netherlands, the ball passed through the bones of the face and brought him to the ground. In the instant that preceded stupefaction, he was able to frame the notion that the ceiling of the room had fallen and crushed him.

The cannon shot which plunged into the brain of Charles XII., did not prevent him from seizing his sword by the hilt. The idea of an attack, and the necessity for defence, was pressed on him by a blow which we should have supposed too tremendous to leave an interval for thought. But it by no means follows that the inflicting of fatal violence is accomplished by a pang. From what is known of the first effect of gun-shot wounds, it is probable that the impression is rather stunning than acute. Unless death be immediate, the pain is as varied as the nature of the injuries, and these are past counting up.

But there is nothing singular in the dying sensation, though Lord Byron remarked the physiological peculiarity that the expression is invariably that of languor; while in death from a stab, the countenance reflects the traits of natural character, of gentleness or ferocity, to the last breath.

Some of these cases are of interest to show with what slight disturbance life may go under a mortal wound, till it finally comes to a sudden stop. A foot soldier at Waterloo, pierced by a musket ball in the hip, begged water of a trooper, who chanced to possess a canteen of beer. The wounded man drank, returned his heartiest thanks, mentioned that his regiment was nearly exterminated, and having proceeded a dozen yards on his way to the rear, fell to the earth, and with one convulsive movement of his limbs, concluded his career.

It should be known to every mother that "Van Deren's Remedy" is just as effectual in curing those irregularities of the bowels of children to which they are subject at all seasons—whether of constipation or diarrhoea—as it is in curing the more dreaded bowel troubles of the summer season. Price, 50 cents. Hall & Ruckel, New York.

